# Self-rated health according to sex and associated factors in Manaus, Brazil, 2019: a population-based cross-sectional study

**DOI:** 10.1590/S2237-96222024V33E2023154.EN

**Published:** 2024-01-22

**Authors:** Isabella Bagni Nakamura, Marcus Tolentino Silva, Taís Freire Galvão

**Affiliations:** 1 Universidade Estadual de Campinas, Programa de Pós-Graduação em Ciências Farmacêuticas, Campinas, SP, Brazil; 2 Universidade de Brasília, Departamento de Saúde Coletiva, Brasília, DF

**Keywords:** Self-Assessment, Gender Inequality, Cross-Sectional Studies, Chronic Disease, Food Insecurity, Autoevaluación, Desigualdad de Género, Estudios Transversales, Enfermedades crónicas, Inseguridad alimentaria, Autoavaliação, Iniquidade de Gênero, Estudos Transversais, Doenças Crônicas, Insegurança Alimentar

## Abstract

**Objective:**

To assess the prevalence and factors associated with poor self-rated health according to respondents’ sex in Manaus, Brazil.

**Methods:**

This was a cross-sectional population-based study with adults in Manaus in 2019. Adjusted prevalence ratios and 95% confidence intervals (95%CI) were calculated using Poisson regression following a hierarchical model.

**Results:**

Poor self-rated health occurred in 35.2% (95%CI 33.3;37.2) of the 2,321 participants and was higher in females (PR = 1.27; 95%CI 1.13;1.43). In the general population, among both sexes, poor self-rated health was higher among the oldest, those with moderate and severe food insecurity and with chronic diseases (p-value < 0.05). Among females, poor health was also higher among the evangelical and those with mild food insecurity. Among males, self-rated health was also poorer among the retired and those with education below elementary level (p-value < 0.001).

**Conclusion:**

The female sex had the poorest health rating, influenced by morbidity and access to food.

## Study contributions

**Main results:**

In 2019, a third of people in Manau self-rated their health as poor, with a higher frequency being found in female sex, the elderly, unemployed, retired, evangelical, obese, those with less schooling, food insecurity and multimorbidity.

**Implications for services:**

Greater attention should be paid to the health status of women in Manaus; they have poorer self-rated health, especially in situations of restricted access to food and presence of chronic diseases.

**Perspectives:**

Future research investigating self-reported negative health rating using clinical or laboratory data may provide better evidence on the magnitude of the problem in the general population.

## Resumen

**Objetivo:**

Analizar la prevalencia y los factores asociados a la mala autoevaluación de salud según sexo en Manaus, Brasil.

**Métodos:**

Se trata de un estudio poblacional transversal con adultos residentes en Manaus en 2019. Las razones de prevalencia ajustadas (RP) y los intervalos de confianza del 95% (IC_95%_) se calcularon mediante regresión jerárquica de Poisson.

**Resultados:**

Autoevaluación mala de salud ocurrió en 35,2% (IC_95%_ 33,3;37,2) de los 2.321 participantes y fue mayor en el sexo femenino (RP = 1,27; IC_95%_1,13;1,43). En la población general, femenina y masculina, la mala autoevaluación de salud fue mayor entre ancianos, con inseguridad alimentaria moderada y grave y con enfermedades crónicas (p-valor < 0,05). En el sexo femenino, la mala salud fue mayor en evangélicas y con inseguridad alimentaria leve. En el sexo masculino, jubilados y con educación inferior al nivel básico también tuvieron una peor autoevaluación (p-valor < 0,001).

**Conclusión:**

Personas de sexo femenino tuvieron una peor valoración de salud, influenciada por la morbilidad y el acceso a la alimentación.

## Resumo

**Objetivo:**

Analisar a prevalência e fatores associados à autoavaliação de saúde ruim segundo o sexo em Manaus.

**Métodos:**

Trata-se de estudo transversal de base populacional com adultos residentes em Manaus em 2019. Razões de prevalências (RP) ajustadas e intervalos de confiança de 95% (IC_95%_) foram calculadas por regressão de Poisson hierarquizada.

**Resultados:**

Saúde autoavaliada como ruim ocorreu em 35,2% (IC_95%_ 33,3;37,2) dos 2.321 participantes e foi maior no sexo feminino (RP = 1,27; IC_95%_ 1,13;1,43). Na população geral, em ambos os sexos, saúde autoavaliada como ruim foi maior entre os mais velhos, com insegurança alimentar moderada e grave e com presença de doenças crônicas (p-valor < 0,05). No sexo feminino, saúde ruim foi maior em evangélicas e com insegurança alimentar leve. No masculino, aposentados e com nível de ensino inferior ao fundamental também apresentaram pior autoavaliação (p-valor < 0,001).

**Conclusão:**

Pessoas do sexo feminino apresentaram pior avaliação de saúde, influenciada por morbidade e acesso a alimentação.

## INTRODUCTION

Self-rated health status is a dynamic measurement. Capable of predicting mortality in populations, an individual’s self-rating of their health needs to be monitored periodically.^1^ As it is a self-reported measurement, it estimates different personal dimensions that help to assess health status. When answering the question about how they rate their health status, the individual begins a particular and subjective process that is dependent on environmental, social and cultural conditions,^2^ a set of physical and psychological factors that are more comprehensive than those obtained through isolated laboratory or clinical examinations. Thus, answers that are more reflexive than automatic emerge.^3^ Overall self-rating of health can identify those who are in a situation of greater vulnerability and with greater health needs.^4^

Women consistently present poorer self-rated health than men: in 28 out of 33 high-income countries, from 2011 to 2015, poor self-rated health was more common in women than in men, in addition to the unemployed. and elderly people.^5^ In Brazil, in 2008, poor or very poor self-rated health was reported by 25% of men and 30% of women; in 2013, by 29% of men and 36% of women;^5^ and in 2019, by 30% of men and 38% of women.^6^

Women living in the North and Northeast regions of Brazil are more likely to perceive their health as poor; and to have higher prevalence of health problems, such as obesity.^7^ In more vulnerable regions and developing countries, it has been demonstrated that the effects of these inequities, inherent to sex, can be more pronounced.^8^

Data on the epidemiological profile of poor self-rated health in the adult population, especially in the North of Brazil, based on analyses segregated by sex, could help to provide an understanding of this dynamic in the population.

The objective of this study was to assess prevalence and factors associated with poor self-rated health according to sex, in the adult population of Manaus, Amazonas, Brazil, in 2019.

## METHODS

*Study design*

This was an analysis of a population-based cross-sectional study, carried out in 2019, with adults living in the city of Manaus.^9^

*Setting*

Manaus is the capital city of Brazil’s largest Federative Unit. In 2021, its population was estimated at 2,255,903 inhabitants;^10^ over half of whom were female according to the 2010 Demographic Census.^11^ Average monthly income of women who worked was 29% lower than that of men. Women living in Manaus accounted for more than half of the school dropouts in the state capital, also in 2010.^11^

*Participants*

Adults (≥ 18 years old) living in Manaus were included in the study, selected through probabilistic sampling in three stages: census tracts; households; and individuals. In the first stage, 250 of the municipality’s 2,461 census tracts were randomly selected, so that in the second stage 20 households per census tract were systematically selected. In the event of refusal, the next household immediately to the right was visited; if refusal occurred again in this case, the next household immediately to the left was visited. In the third stage, all household residents present were registered on an electronic device, and one resident was selected to answer the questionnaire, considering sex and age quotas.^9^

*Variables*

Once the dependent variable “self-rated health” (good; poor) had been defined, the independent variables were included:

a) sex (male; female);

b) age group (at last birthday: 18-24; 25-34; 35-44; 45-59; 60 or over);

c) race/skin color (White; Black);

d) occupation (formal work; informal work; retired; student/housewife; unemployed);

e) economic classification (A/B; C; D/E, where A corresponds to the wealthiest; and E corresponds to the poorest);

f) schooling (higher education or above; high school education; elementary education; below elementary);

g) religion (catholic; evangelical; other; none);

h) food insecurity (none; mild; moderate; severe);

i) body composition (eutrophy; overweight; obesity); and

j) number of chronic dieases (none; one; two or more).

*Data source and measurement*

The main outcome of the study was measured using the question “*What is your overall health status?”*, to which interviewees could answer “very good”, “good”, “regular”, “poor” or “very poor”. The five answer options were consolidated into two categories: (i) good health, bringing together those who responded “very good” or “good”; and (ii) poor health, for those who chose the answer option “regular”, “poor” or “very poor”.

Food insecurity was measured using the Brazilian Food Insecurity Scale (*Escala Brasileira de Insegurança Alimentar* - EBIA), consisting of 14 questions involving dimensions such as concern and anguish due to the possibility of lack of food, strategies for using less food and the experience of going hungry, in a three-month recall period.^12^ Food insecurity was classified according to the score obtained, as none (0), mild (1-3), moderate (4-5) or severe (6-8), in households with no children (< 18 years). In households with children, the classification was based on the following scores: none (0), mild (1-5), moderate (6-9) or severe (10-14).^12^ Body composition was measured by the body mass index (BMI), that is, the ratio of weight (in kilograms) to height (in meters) squared, both self-reported by the participants: BMI ≤ 24.9 kg/m² was classified as eutrophy; BMI between 25.0 and 29.9 kg/m² as overweight; and BMI ≥ 30 kg/m² as obesity.

Economic classification was measured according to the 2018 Brazilian Economic Classification Criteria (*Critério Brasil de Classificação Econômica*), based on the education level of the head of the family, presence of household appliances and urbanization surrounding the home.^13^ Race/skin color was self-reported by each participant as White, Black (Black and Brown), Asian or Indigenous, and then dichotomized into White (White and Asian) and Black (Black and Indigenous).

The number of chronic diseases was obtained from self-reported information on previous diagnosis of hypertension, diabetes, high cholesterol, heart disease, stroke, asthma, arthritis/rheumatism, back problem, depression, schizophrenia, bipolar disorder, psychosis, obsessive-compulsive disorder, pulmonary emphysema, chronic bronchitis, chronic obstructive pulmonary disease, cancer and chronic kidney failure. The question about self-rating of health status was asked before the questions about presence of chronic diseases.

Previously trained interviewers collected participants’ ansers, with pre-configured questionnaires using the SurveyToGo software (Dooblo Ltd, Israel), on mobile devices (Intel TabPhone 710 Pro). The completed questionnaires were synchronized with the database via internet.

A sample of 150 people answered the questionnaire before it was officially asministered, with the purpose of validating understanding of the questions. The group that participated in this pre-test was also included in the final sample of the study, as the initial questionnaire approved in the pre-test was not modified. The question about self-rated health status precedes the questions about chronic diseases. In order to ensure the reliability of data collection, 20% of the interviewees had their answers audited by telephone.^9^

*Study size*

The sample size was estimated at 2,300 participants. This calculation considered an estimated 20% demand for health services in the last 15 days, considering a 95% confidence interval (95%CI), design effect of 1.5 and an estimated population of 2,106,322 inhabitants living in Manaus, in 2018.^14^

*Statistical analysis*

Prevalence of poor self-rated health was estimated for all study participants, and for females and males separately, according to the independent variables. Differences were submitted to Pearson’s chi-square test, with a 5% significance level. Poisson regression with robust variance and 95%CI was used to calculate the unadjusted and adjusted prevalence ratios (PRs) of poor self-rated health, according to the independent variables, in separate models for the general population, females and males, using hierarchical analysis, considering variables proximal and distal to poor self-rated health.^15^ Sociodemographic variables with p-value ≤ 0.20 in the unadjusted analysis comprised the first level. The second level of analysis included health behavior variables (food insecurity and body composition), plus the significant variables from the first level. The third level was composed of chronic diseases plus significant variables from the previous levels. Associations with p-value < 0.05 were considered statistically significant.

Probability of poor self-rated health stratified by sex, according to food insecurity levels and religion was illustrated graphically.

Data analysis was performed using Stata 14.2 software (StataCorp LP, College Station, Texas, United States), using the survey module (svy) to consider the complex design employed.

*Ethical aspects*

The study project was approved by the Human Research Ethics Committee, of the *Universidade Federal do Amazonas*: Opinion No. 3.102.942, dated December 28, 2018 [Certificate of Submission for Ethical Appraisal (*Certificado de Apresentação para Apreciação Ética*) No. 04728918.0.0000.5020]. All participants signed a free and informed Consent form prior to the interview.

## RESULTS

**Figure 1:**
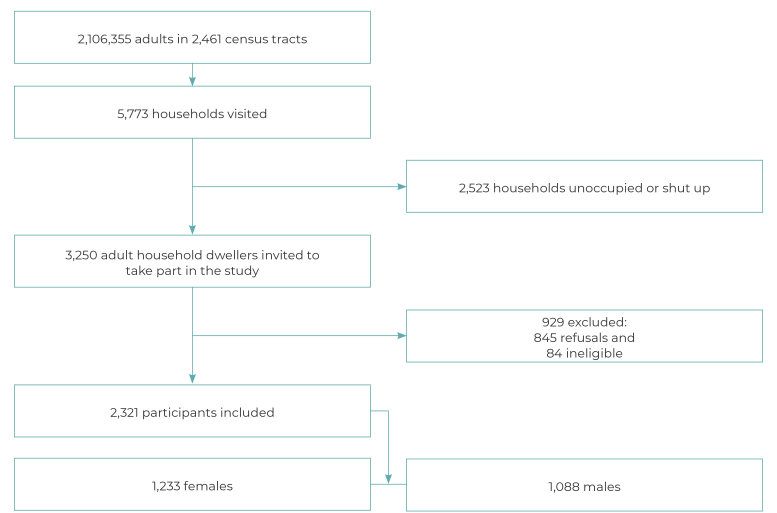
Participant recruitment process, Manaus, Brazil, 2019

A total of 2,321 adults were included in the study (Figure 1), the majority of whom were female (52.2%), of Black race/skin color (85.0%) and economic classification C (53.6%). Regarding age, 25.1% were between 25 and 34 years old; 41.1% were unemployed; and 45.4% practiced an evangelical religion. Just over half had completed high school education (50.4%) (Table 1). In female sex, 56.3% were unemployed, while this percentage was 24.4% for the male sex. The majority of females practiced an evangelical religion (51.6%); while for males the Catholic religion was the most practiced, by 43.4% of them (Supplementary Table 1).

**Table 1:** Characteristics of the participants (N = 2,321) and prevalence of poor self-rated heath, Manaus, Brazil, 2019

Variables	Total N (%)	Poor health N (%)	p-value
Sex			< 0.001
Male	1,088 (47.8)	322 (29.3)	
Female	1,233 (52.2)	501 (40.7)	
Age group (years)			< 0.001
18-24	405 (19.3)	105 (25.6)	
25-34	586 (25.1)	160 (27.2)	
35-44	553 (22.8)	177 (31.9)	
45-59	526 (21.2)	242 (45.9)	
≥ 60	251 (11.6)	139 (55.5)	
Race/skin color			0.745
White	349 (15.0)	121 (34.5)	
Black	1,972 (85.0)	702 (35.4)	
Occupation			< 0.001
Formal work	419 (17.9)	110 (26.2)	
Informal work	661 (28.1)	222 (33.3)	
Retired	162 (7.2)	90 (55.4)	
Student/housewife	124 (5.7)	30 (23.7)	
Unemployed	955 (41.1)	371 (38.6)	
Economic classification			< 0.001
A/B	282 (12.3)	83 (29.2)	
C	1,244 (53.6)	414 (33.0)	
D/E	795 (34.1)	326 (40.8)	
Schooling			< 0.001
Higher education or above	153 (6.5)	42 (27.3)	
High school education	1,171 (50.4)	338 (28.5)	
Elementary education	432 (18.9)	159 (36.7)	
Below elementary	565 (24.2)	284 (50.2)	
Religion			0.081
Catholic	931 (39.9)	317 (33.9)	
Evangelical	1,054 (45.4)	401 (37.8)	
Other	137 (5.9)	43 (31.4)	
None	199 (8.8)	62 (30.5)	
Food insecurity^a^			< 0.001
None	777 (33.8)	204 (26.2)	
Mild	874 (37.7)	286 (32.4)	
Moderate	319 (13.8)	153 (47.9)	
Severe	345 (14.7)	179 (51.4)	
Body composition			0.002
Eutrophy	971 (42.6)	322 (32.9)	
Overweight	792 (33.8)	296 (33.7)	
Obesity	558 (23.6)	232 (41.6)	
No. of chronic diseases			< 0.001
0	921 (40.1)	164 (17.8)	
1	682 (29.3)	218 (31.8)	
≥ 2	718 (30.6)	441 (61.4)	

a) Data missing for 6 participants.

Prevalence of poor self-rated health was 35.2% (95%CI 33.3;37.2) in the general population, 40.7% (95%CI 37.9;43.4) for the female sex and 29.3% (95%CI 26.6;32.0) for the male sex (data not shown).

17.0% of female participants were in a situation of severe food insecurity, compared to 12.4% of males. Poor self-rated health was reported by females aged 60 or over (60.0%), the unemployed (42.1%), those with severe food insecurity (59.6%) and those who had two or more chronic diseases (61.2%). Poor health occurred more among people who were retired (54.6%), had no education (46.3%) and had been diagnosed as having two or more chronic diseases (61.8%) (Supplementary Table 1).

Poor self-rated health was greater among female sex (PR = 1.27; 95%CI 1.13;1.43); older people (35-44 years-old (PR = 1.26; 95%CI 1.01;1.56); 45-59 years-old (PR = 1.78; 95%CI 1.45;2.19); 60 years-old or over (PR = 1.85; 95%CI 1.46;2.34); unemployed (PR = 1.23; 95%CI 1.02;1.49); retirees (PR = 1.29; 95%CI 1.02;1.64); no education (PR = 1.45; 95%CI 1.09;1.92); practitioners of evangelical religion (PR = 1.17; 95%CI 1.04;1.31); those with mild food insecurity (PR = 1.22; 95%CI 1.05;1.41), moderate food insecurity (PR = 1.70; 95%CI 1.45;1.99) and severe food insecurity (PR = 1.67; 95%CI 1.43;1.95); obese people (PR = 1.16; 95%CI 1.01;1.32); and with one (PR = 1.71; 95%CI 1.44;2.04) or more (PR = 2.90; 95%CI 2.47;3.40) chronic diseases (Table 2).

**Table 2:** Poor self-rated health unadjusted and adjusted prevalence ratios (PR) and 95% confidence intervals (95%CI), according to the study variables (N = 2,321), Manaus, Brazil, 2019

Variables	Unadjusted PR (95%CI)	p-value	Adjusted PR (95%CI)	p-value
First level				
Sex		< 0.001		< 0.001
Male	1.00		1.00	
Female	1.39 (1.24;1.56)		1.27 (1.13;1.43)	
Age group (years)		< 0.001		< 0.001
18-24	1.00		1.00	
25-34	1.06 (0.86;1.31)		1.10 (0.88;1.36)	
35-44	1.25 (1.02;1.53)		1.26 (1.01;1.56)	
45-59	1.79 (1.48;2.17)		1.78 (1.45;2.19)	
≥ 60	2.17 (1.78;2.64)		1.85 (1.46;2.34)	
Race/skin color		0.746		
White	1.00			
Black	1.03 (0.88;1.20)			
Occupation		< 0.001		0.057
Formal work	1.00		1.00	
Informal work	1.27 (1.05;1.54)		1.06 (0.87;1.29)	
Retired	2.11 (1.71;2.62)		1.29 (1.02;1.64)	
Student/housewife	0.91 (0.64;1.29)		1.02 (0.70;1.48)	
Unemployed	1.47 (1.23;1.77)		1.23 (1.02;1.49)	
Economic classification		< 0.001		0.600
A/B	1.00		1.00	
C	1.13 (0.93;1.38)		0.99 (0.81;1.20)	
D/E	1.40 (1.15;1.71)		1.05 (0.85;1.29)	
Schooling		< 0.001		< 0.001
Higher education or above	1.00		1.00	
High school education	1.05 (0.79;1.37)		1.05 (0.80;1.38)	
Elementary education	1.34 (1.01;1.79)		1.34 (1.00;1.79)	
Below elementary	1.84 (1.40;2.42)		1.45 (1.09;1.92)	
Religion		0.085		0.060
Catholic	1.00		1.00	
Evangelical	1.12 (0.99;1.26)		1.17 (1.04;1.31)	
Other	0.93 (0.71;1.21)		1.05 (0.81;1.37)	
None	0.90 (0.72;1.13)		1.14 (0.91;1.44)	
Second level				
Food insecurity		< 0.001		< 0.001
None	1.00		1.00	
Mild	1.24 (1.06;1.44)		1.22 (1.05;1.41)	
Moderate	1.83 (1.55;2.16)		1.70 (1.45;1.99)	
Severe	1.96 (1.68;2.30)		1.67 (1.43;1.95)	
Body composition		0.001		0.067
Eutrophy	1.00		1.00	
Overweight	1.02 (0.90;1.17)		1.02 (0.90;1.16)	
Obesity	1.27 (1.11;1.45)		1.16 (1.01;1.32)	
Third level				
No. of chronic diseases		< 0.001		< 0.001
0	1.00		1.00	
1	1.79 (1.50;2.14)		1.71 (1.44;2.04)	
≥ 2	3.46 (2.97;4.02)		2.90 (2.47;3.40)	

Among the female sex, poor self-rated health was higher in the following variables-categories: age 45-59 (PR = 1.75; 95%CI 1.37;2.25) and 60 years or over (PR = 1.83; 95%CI 1.39;2.42); evangelical (PR = 1.26; 95%CI 1.09;1.46); those with mild food insecurity (PR = 1.33; 95%CI 1.09;1.62), moderate food insecurity (PR = 1.74; 95%CI 1.40;2.15) and severe food insecurity (PR = 1.87; 95%CI 1.52;2.30); and with one (PR = 1.61; 95%CI 1.29;2.00) or two or more (PR = 2.37; 95%CI 1.94;2.89) chronic diseases.

Among the male sex, poor self-rated health was higher in individuals aged 45-59 years (PR = 1.75; 95%CI 1.23;2.50) and 60 years or over (PR = 1.78; 95%CI 1.17;2.71); retirees (PR = 1.42; 95%CI 1.01;2.02); no formal education (PR = 1.94; 95%CI 1.15;3.29); with moderate food insecurity (PR = 1.69; 95%CI 1.31;2.18) and severe food insecurity (PR = 1.44; 95%CI 1.10;1.87); and with one (PR = 1.80; 95%CI 1.36;2.39) or two or more (PR = 3.84; 95%CI 2.97;4.90) chronic diseases (Table 3).

**Table 3:** Poor self-rated health unadjusted and adjusted prevalence ratios (PR) and 95% confidence intervals (95%CI), according to the study variables, by sex, Manaus, Brazil, 2019

Variables	Female sex (N = 1,233)				Male sex (N = 1,088)			
	Unadjusted PR (95%CI)	p-value	Adjusted PR (95%CI)	p-value	Unadjusted PR (95%CI)	p-value	Adjusted PR (95%CI)	p-value
First level								
Age group (years)		< 0,001		< 0.001		< 0.001		0.005
18-24	1.00		1.00		1.00		1.00	
25-34	1.01 (0.79;1.31)		1.04 (0.80;1.35)		1.13 (0.78;1.63)		1.16 (0.79;1.69)	
35-44	1.08 (0.84;1.39)		1.13 (0.86;1.47)		1.51 (1.07;2.13)		1.45 (1.00;2.10)	
45-59	1.66 (1.33;2.08)		1.75 (1.37;2.25)		1.97 (1.42;2.75)		1.75 (1.23;2.50)	
≥ 60	1.88 (1.48;2.39)		1.83 (1.39;2.42)		2.57 (1.82;3.63)		1.78 (1.17;2.71)	
Race/skin color		0.688				0.513		
White	1.00		1.00		1.00		1.00	
Black	0.96 (0.80;1.16)				1.09 (0.84;1.42)			
Occupation		0.002		0.339		< 0.001		
Formal work	1.00		1.00		1.00		1.00	0.127
Informal work	1.26 (0.94;1.71)		1.06 (0.78;1.44)		1.22 (0.94;1.57)		1.03 (0.80;1.34)	
Retired	1.82 (1.31;2.53)		1.18 (0.83;1.68)		2.25 (1.70;2.98)		1.42 (1.01;2.02)	
Student/housewife	1.03 (0.67;1.59)		1.19 (0.75;1.88)		0.60 (0.31;1.18)		0.76 (0.37;1.53)	
Unemployed	1.36 (1.03;1.79)		1.24 (0.93;1.64)		1.23 (0.93;1.62)		1.21 (0.92;1.60)	
Economic classification		0.037		0.722		0.008		0.454
A/B	1.00		1.00		1.00		1.00	
C	1.18 (0.90;1.54)		1.10 (0.84;1.44)		1.02 (0.76;1.37)		0.86 (0.64;1.16)	
D/E	1.35 (1.03;1.77)		1.12 (0.85;1.49)		1.37 (1.01;1.85)		0.96 (0.70;1.31)	
Schooling		< 0.001		0.010		< 0.001		0.002
Higher education or above	1.00		1.00		1.00		1.00	
High school education	1.01 (0.74;1.39)		0.96 (0.69;1.32)		1.19 (0.71;1.98)		1.25 (0.75;2.07)	
Elementary education	1.32 (0.95;1.84)		1.23 (0.88;1.72)		1.47 (0.86;2.52)		1.60 (0.93;2.74)	
Below elementary	1.61 (1.17;2.20)		1.23 (0.88;1.70)		2.38 (1.43;3.95)		1.94 (1.15;3.29)	
Religion		0.067		0.016		0.887		
Catholic	1.00		1.00		1.00		1.00	
Evangelical	1.18 (1.02;1.37)		1.26 (1.09;1.46)		0.94 (0.76;1.15)			
Other	1.01 (0.71;1.45)		1.08 (0.75;1.56)		0.89 (0.60;1.30)			
None	0.88 (0.64;1.21)		1.11 (0.80;1.54)		0.94 (0.68;1.30)			
Second level								
Food insecurity		< 0.001		< 0.001		< 0.001		< 0.001
None	1.00		1.00		1.00		1.00	
Mild	1.35 (1.09;1.65)		1.33 (1.09;1.62)		1.06 (0.84;1.34)		1.10 (0.88;1.38)	
Moderate	1.87 (1.50;2.33)		1.74 (1.40;2.15)		1.68 (1.29;2.18)		1.69 (1.31;2.18)	
Severe	2.14 (1.75;2.63)		1.87 (1.52;2.30)		1.57 (1.20;2.05)		1.44 (1.10;1.87)	
Body composition		0.079		0.359		0.061		0.167
Eutrophy	1.00		1.00		1.00		1.00	
Overweight	0.99 (0.84;1.17)		0.99 (0.84;1.16)		1.09 (0.88;1.35)		1.04 (0.85;1.28)	
Obesity	1.17 (1.00;1.37)		1.10 (0.94;1.28)		1.32 (1.05;1.67)		1.24 (0.98;1.57)	
Third level								
No. of chronic diseases		< 0.001		< 0.001		< 0.001		< 0.001
0	1.00		1.00		1.00		1.00	
1	1.67 (1.33;2.09)		1.61 (1.29;2.00)		1.90 (1.43;2.53)		1.80 (1.36;2.39)	
≥ 2	2.75 (2.27;3.33)		2.37 (1.94;2.89)		4.44 (3.49;5.65)		3.84 (2.97;4.90)	

**Figure 2:**
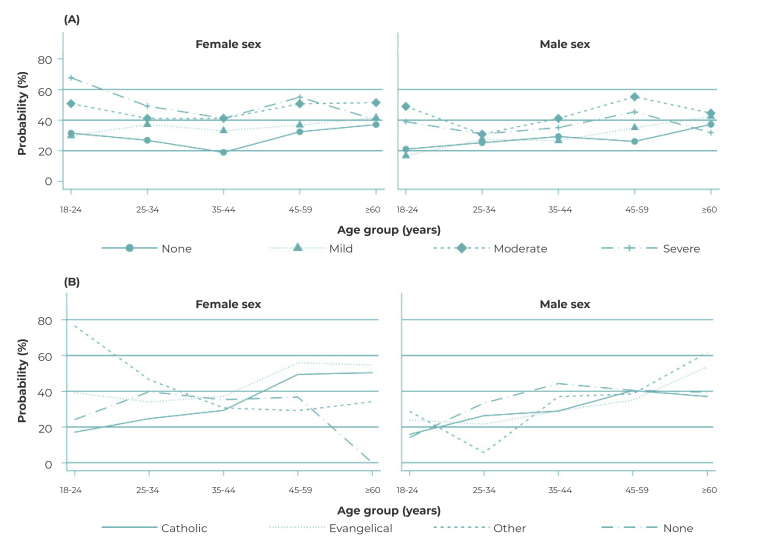
Probabilities of poor self-rated health among adults by age group and sex, according to level of food insecurity (A) and religion (B), Manaus, Brazil, 2019

Poor self-rated health was higher among females than among males, at all levels of food insecurity, with greater probability of poor self-rated health for females, both younger and elderly (Figure 2A). People of the female sex aged 60 or over, who practiced an evangelical religion, had a higher probability of poor self-rated health than people of the male sex in the same age group (Figure 2B).

## DISCUSSION

Poor self-rated health affected one in three adults in Manaus. Poor self-rated health was greater among the female sex, elderly people, retired people, those who were unemployed, those with a lower level of schooling, practitioners of an evangelical religion, at all levels of food insecurity, and with two or more illnesses. Females of evangelical religion, at all levels of food insecurity and with chronic diseases had poorer self-rated health. Among the male sex, moderate food insecurity and the presence of chronic diseases significantly increased perception of poor health.

Our research was based on self-reported information, subject to information bias. Recall bias may also have interfered with self-rated health, as participants could have forgotten negative events that occurred over the period. Factors inherent to the cross-sectional design are also limiting, given the impossibility of attesting causality and establishing temporal relationships. The real magnitude of poor self-rated health in the population may be over or underestimated, due to survival bias, as healthier individuals have longer survival; and selection bias, considering the possibility of individuals with poor health status not being at home due to this condition.

Self-rated health is a valid indicator for objective health measurements and a strong predictor of mortality,^1^ ^),(^ ^4^ in addition to being subject to the influence of cultural and social factors on the understanding of health.^16^ The data were collected in 2019 and may not reflect the food insecurity scenario resulting from the COVID-19 pandemic, which saw an increase in hunger in Brazil. Our research used a probabilistic sample, allowing representation of the adult population of Manaus. In the questionnaire, the question about self-rated health was placed before questions about chronic diseases, helping to minimize the influence of remembering health problems on overall health status rating.

Prevalence of poor self-rated health reported in this study was higher than the national prevalence rate (34%), found in a study conducted with data from the 2013 National Health Survey (*Pesquisa Nacional de Saúde* - PNS), which included 59,758 adults.^7^ The high prevalence of poor health in Manaus is consistent with an analysis made of the Chronic Disease Risk and Protective Factors Surveillance Telephone Survey (*Vigilância de Fatores de Risco e Proteção para Doenças Crônicas por Inquérito Telefônico* - VIGITEL) from 2011 to 2020, which found that average prevalence of poor self-rated health was 3.9% in the Northern region, 3.5% in the Midwest and Northeast regions, 3.0% in the Southeast and 2.9% in the Southern region of Brazil.^17^ That analysis measured self-rated health dichotomized into good (“very good”, “good” and “regular”) and poor (“poor” and “very poor”).^17^ National analysis of the 2006 VIGITEL found poor health in 5.4% of the population, following the same categorization.^18^ In 2020 another study based on the dichotomization of health into good (“very good” and “good”) and poor (“regular”, “poor” and “very poor”),^19^ similarly to our research, found poor health in 28.8% of Brazilians. These changes in forms of measurement may explain the variation in results between studies.

Poor self-rated health and food insecurity were more frequent in females than in males. Sex differences in these outcomes are consistent with national and international data, revealing worse results among women.^7^ ^),(^ ^20^ Informal work is more frequent among women, such as domestic employment and self-employment. They receive lower salaries than men, for the same jobs. Moreoever, they are mainly responsible for domestic and child care, in addition to working, which greatly affects their health.^21^ Income is the main determinant of food insecurity,^22^ and the inequities inherent to sex, which women face, worsen this outcome. They are often blamed for lack of food in their homes, taking on preparing food and feeding the family as a female responsibility.^23^

Food insecurity, at all levels, worsened self-rated health in the general population. females with mild food insecurity (uncertainty regarding access to food or the quality of food), moderate food insecurity (insufficient quantities of food) and severe food insecurity (food deprivation and hunger) had poorer self-rated health. Among males, moderate food insecurity was associated with poorer self-rated health. Nutritional deficiency caused by food insecurity increases the risk of obesity and leads to the development of acute and chronic morbidities, worsening quality of life and the way a person rates their health status.^24^ ^),(^ ^25^

In the general population, poor self-rated health was greater in those aged over 35, while for males and females separately it was greater with effect from 45 years of age. Increasing age is accompanied by greater prevalence of comorbidities and functional disabilities, which may explain the relationship with poorer rating of one’s own health status.

Lower levels of schooling worsened self-rated health in the general population and among males. Among females, level of education was not associated with poor self-rated health. Analysis of the 2013 PNS^7^ identified a worsening gradient in the self-rated health of 59,758 participants as schooling decreased, possibly explained by less access to information, health services and better living conditions.

Presence of chronic diseases worsened self-rated health in the general population, as well as among males and among females. Chronic diseases are major factors regarding illness and mortality among the population. Analysis of the 2013 PNS, which included 11,697 elderly people, found greater multimorbidity among women, those who were older and those with lower schooling.^26^ Despite women having higher prevalence of chronic diseases,^27^ diagnosis of at least one chronic disease was sufficient for self-rated health status to be poorer for both sexes in our study.

Poor self-rated health was more frequent among evangelicals in the general population and among females. There was no association between religious practice and worsening of self-rated health among males, in addition, the majority of individuals who did not practice any religion were male. Analysis of the 2013 PNS with 64,348 individuals found that women seek religious activities more than men.^27^ Negative events in life, including poor health and existing illnesses, can encourage religious practice.^28^

In the general population, obesity was associated with poorer self-rated health, while this was not found in separate analyses according to the sex. Obesity is related to the presence of hypertension, diabetes or chronic non-communicable disease.^29^ In another study also using data from the 2013 PNS, involving 59,402 adults, the odds of a person with obesity having poor self-rated health was 1.3 time greater than that of a person without this condition.^29^

Being retired and unemployed led to poorer self-rated health in the general population, while among males only being retired led to poorer health ratings. These associations did not occur among females. A drop in family income is a determining factor for triggering mental disorders, such as depression and anxiety, due to concerns about one’s own livelihood and that of one’s family, for example.^30^ Unemployment was also associated with poorer self-rated health in the 2013 PNS, being considered a determining factor for this indicator.^7^

In conclusion, poor self-rated health occurred in approximately four out of every ten adults living in Manaus, and was greater among socioeconomically vulnerable groups. Policies that guarantee access and equity regarding basic rights, including healthy food and a job market with equal rights between men and women, will potentially reduce situations that lead to the poor rating of health status found among inhabitants of Manaus.

## Supplementary Table 1

### - Characteristics of the participants and prevalence of poor self-rated health stratified by sex, Manaus, Brazil, 2019

**Supplementary Table:** 1 - Characteristics of the participants and prevalence of poor self-rated health stratified by sex, Manaus, Brazil, 2019

Variables	Female sex (N = 1,233)			Male sex (N = 1,088)		
	Total n (%)	Poor health n (%)	p-value	Total n (%)	Poor health n (%)	p-value
Age group (years)			< 0.001			< 0.001
18-24	213 (18.5)	68 (31.9)		192 (20.1)	37 (19.3)	
25-34	306 (24.8)	99 (32.4)		280 (25.5)	61 (21.8)	
35-44	295 (22.9)	102 (34.6)		258 (22.8)	75 (29.1)	
45-59	279 (21.2)	148 (53.1)		247 (21.1)	94 (38.1)	
≥ 60	140 (12.6)	84 (60.0)		111 (10.5)	55 (49.6)	
Race/skin color			0.691			0.507
White	174 (14.1)	73 (42.0)		175 (16.1)	48 (27.2)	
Black	1,059 (85.9)	428 (40.4)		913 (83.9)	274 (29.7)	
Occupation			0.003			< 0.001
Formal work	126 (10.0)	39 (30.9)		293 (26.6)	71 (24.2)	
Informal work	268 (21.3)	105 (39.1)		393 (35.5)	117 (29.5)	
Retired	76 (6.5)	43 (56.2)		86 (7.9)	47 (54.6)	
Student/housewife	69 (5.9)	22 (31.8)		55 (5.6)	8 (14.5)	
Unemployed	694 (56.3)	292 (42.1)		261 (24.4)	79 (29.8)	
Economic classification			0.033			0.009
A/B	126 (10.2)	42 (33.2)		156 (14.4)	41 (26.0)	
C	651 (52.9)	255 (39.2)		593 (54.4)	159 (26.5)	
D/E	456 (36.9)	204 (44.9)		339 (31.2)	122 (35.6)	
Schooling			< 0.001			< 0.001
Higher education or above	87 (7.0)	29 (33.3)		66 (5.9)	13 (19.5)	
High school education	605 (48.8)	205 (33.8)		566 (52.2)	133 (23.1)	
Elementary education	230 (18.9)	101 (44.0)		202 (18.9)	58 (28.6)	
Below elementary	311 (25.3)	166 (53.5)		254 (23.0)	118 (46.3)	
Religion			0.059			0.887
Catholic	454 (36.8)	170 (37.5)		477 (43.4)	147 (30.5)	
Evangelical	636 (51.6)	281 (44.2)		418 (38.6)	120 (28.6)	
Other	56 (4.5)	21 (38.1)		81 (7.4)	22 (27.0)	
None	87 (7.1)	29 (33.0)		112 (10.6)	33 (28.7)	
Food insecurity^a^			< 0.001			< 0.001
None	345 (28.2)	95 (27.8)		432 (39.9)	109 (25.0)	
Mild	484 (39.3)	181 (37.4)		390 (35.9)	105 (26.5)	
Moderate	191 (15.5)	99 (52.0)		128 (11.8)	54 (42.0)	
Severe	211 (17.0)	126 (59.6)		134 (12.4)	53 (39.2)	
Body composition			0.089			0.068
Eutrophy	499 (41.0)	194 (39.0)		472 (44.3)	128 (26.8)	
Overweight	392 (31.6)	151 (38.5)		400 (36.2)	118 (29.1)	
Obesity	342 (27.4)	156 (45.7)		216 (19.5)	76 (35.4)	
No. of chronic diseases			< 0.001			< 0.001
0	433 (35.3)	96 (22.3)		488 (45.4)	68 (13.9)	
1	349 (28.2)	129 (37.1)		333 (30.5)	89 (26.5)	
≥ 2	451 (36.5)	276 (61.2)		267 (2)	165 (61.8)	

a) Data missing for 2 participants of the female sex and 4 of the male sex.
